# Prospective validation of a model-informed precision dosing tool for vancomycin treatment in neonates

**DOI:** 10.1128/aac.01591-23

**Published:** 2024-04-05

**Authors:** Riste Kalamees, Hiie Soeorg, Mari-Liis Ilmoja, Kadri Margus, Irja Lutsar, Tuuli Metsvaht

**Affiliations:** 1 Department of Microbiology, University of Tartu, Tartu, Estonia; 2 Pediatric and Neonatal Intensive Care Unit, Tallinn Children’s Hospital, Tallinn, Estonia; 3 Department of Neonatology, East Tallinn Central Hospital, Tallinn, Estonia; 4 Pediatric and Neonatal Intensive Care Unit, Clinic of Anaesthesiology and Intensive Care, Tartu University Hospital, Tartu, Estonia; Providence Portland Medical Center, Portland, Oregon, USA

**Keywords:** vancomycin, neonates, model-informed precision dosing

## Abstract

We recruited 48 neonates (50 vancomycin treatment episodes) in a prospective study to validate a model-informed precision dosing (MIPD) software. The initial vancomycin dose was based on a population pharmacokinetic model and adjusted every 36–48 h. Compared with a historical control group of 53 neonates (65 episodes), the achievement of a target trough concentration of 10–15 mg/L improved from 37% in the study to 62% in the MIPD group (*P* = 0.01), with no difference in side effects.

## INTRODUCTION

Vancomycin is widely used in neonatal intensive care units (NICUs) (1). The pharmacokinetics (PK) of vancomycin is highly variable because of developmental shifts and changing disease severity in neonates. With standard dosing regimens, the therapeutic goal of vancomycin trough concentration (*C*_trough_) 10–15 mg/L is achieved in <30% of neonates (2, 3), but in 71%–94% when applying model-informed precision dosing (MIPD) (4–6). However, clinical studies in neonates remain scarce (6, 7). We aimed to validate our previously described MIPD software for optimizing vancomycin therapy in neonates (8, 9).

A prospective study cohort and a historical control group were recruited from three Estonian NICUs. We included neonates and infants ≤90 days, who received vancomycin between January 2016 and May 2019 (control group) or June 2019 and June 2021 (study group), had ≥1 vancomycin steady-state concentration (SS-C, defined as measurement after ≥36 h of a given regimen) available, and did not receive renal replacement therapy.

Vancomycin was administered as a 60-min infusion. The timing of vancomycin concentration measurement and dose adjustments in the control group were determined by the treating physician. In the study group, the first optimized regimen was calculated with MIPD software (9) based on the vancomycin popPK model by Zhao et al. (10). Vancomycin treatment ≤24 h before the study was allowed. In the following dose optimizations, all available vancomycin concentrations were included. For each predefined dosing interval (6–24 with 2-h intervals), the dose with the highest probability of achieving the target SS-C_trough_ 10–15 mg/L (TA%, target attainment percentage, TA% SS-C_trough 10-15 mg/L_) 36–48 h after implementation was calculated and applied for ≥36 h to reach steady state before the next TDM. After the first 20 patients’ dose was optimized for both SS-C_trough_ and AUC/MIC (the area under the concentration-time curve over 24 h divided by the MIC) targets, short intervals with unacceptably low AUC/MIC were frequently recommended for the highest TA% C_trough 10-15mg /L_. An MIC value of 1 mcg/mL was applied in all calculations.

**Fig 1:**
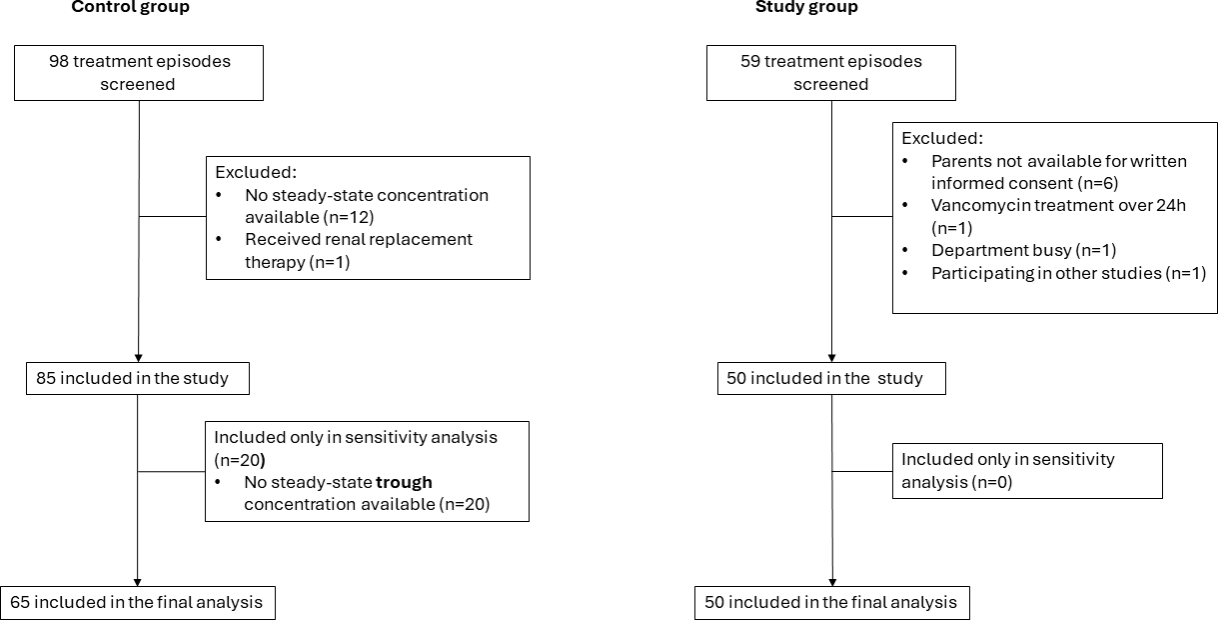
Flowchart presenting the study group formation.

Sample size calculation is presented in the supplemental material. TA% between the groups was compared for three targets: SS-C_trough_ between 10–15, 8–17, and 5–20 mg/L (TA% *C*_trough 10-15/8-17/5-20 mg/L_). All SS-C_trough_ measurements were included in the outcome analysis (Fig. 1). A sensitivity analysis included missing SS-C_trough_ predicted based on TDM results for the same dosing interval using the popPK model by Tseng et al. (11). Logistic or linear regression with generalized estimating equations and exchangeable correlation structure was used to compare the groups.

A total of 48 patients with 50 treatment episodes in the study and 66 with 85 treatment episodes in the control group were recruited (Fig. 1). A total of 115 SS-C_trough_ (defined as measurement up to 1.5 h before the next scheduled infusion) in the study and 95 in the control group were retained in the final analysis (additional 8 and 56 SS-non-C_trough_, respectively, in the sensitivity analysis).

Patients in the study group had significantly lower birth weight, gestational age, and body weight but higher creatinine levels compared with the control group (Table 1).

**TABLE 1:** Demographic characteristics of the study population^*b*^

Study group	Control group (*n* = 66)	Intervention group (*n* = 48)	*P*-value
Treatment episodes (no.)	85	50	
Birth weight (g)	970 (466–4,374)	830 (520–3,472)	0.002
Extremely low BW (<1,000 g), no. (%)	43 (50.6)	34 (68)	
Gestational age at birth (weeks)	27.3 (23–41.1)	26.6 (23–41.3)	0.003
Extremely low GA <28 week, no. (%)	50 (58.8)	33 (66)	
Current weight^*a*^ (g)	1,075 (473–4,830)	905 (436–3,610)	0.001
Postnatal age^*a*^ (days)	10 (1–66)	9.5 (3-69)	NS
Serum creatinine^*a*^ (μmol/L)	47.0 (17–110)	50.0 (4–265)	0.02
Respiratory support, no. (%)			
Spontaneous ventilation (SV)	14 (16.5)	5 (10)	(SV + NIV vs IV)
Non-invasive ventilation (NIV)	36 (42.4)	30 (60)	
Invasive ventilation (IV)	35 (41.2)	15 (30)	NS/NS
Need for inotropes, no. (%)	31 (36.5)	15 (30)	NS/NS

^*a*^ At the start of the treatment; NS, not statistically significant.

^*b*^ Data are presented as median (range) if not stated otherwise.

The administered doses were similar with a median (range) of 22.8 (15.0–67.7) in the study (in two episodes, optimized doses were considered inadequate and not implemented) and 26.9 (12.6–46.1) mg/kg of body weight/day in the control group, respectively. Dosing intervals were significantly shorter in the study vs the control group [median (range) 12 (6–18) h vs 12 (8–24) h; *P* < 0.001].

**Fig 2:**
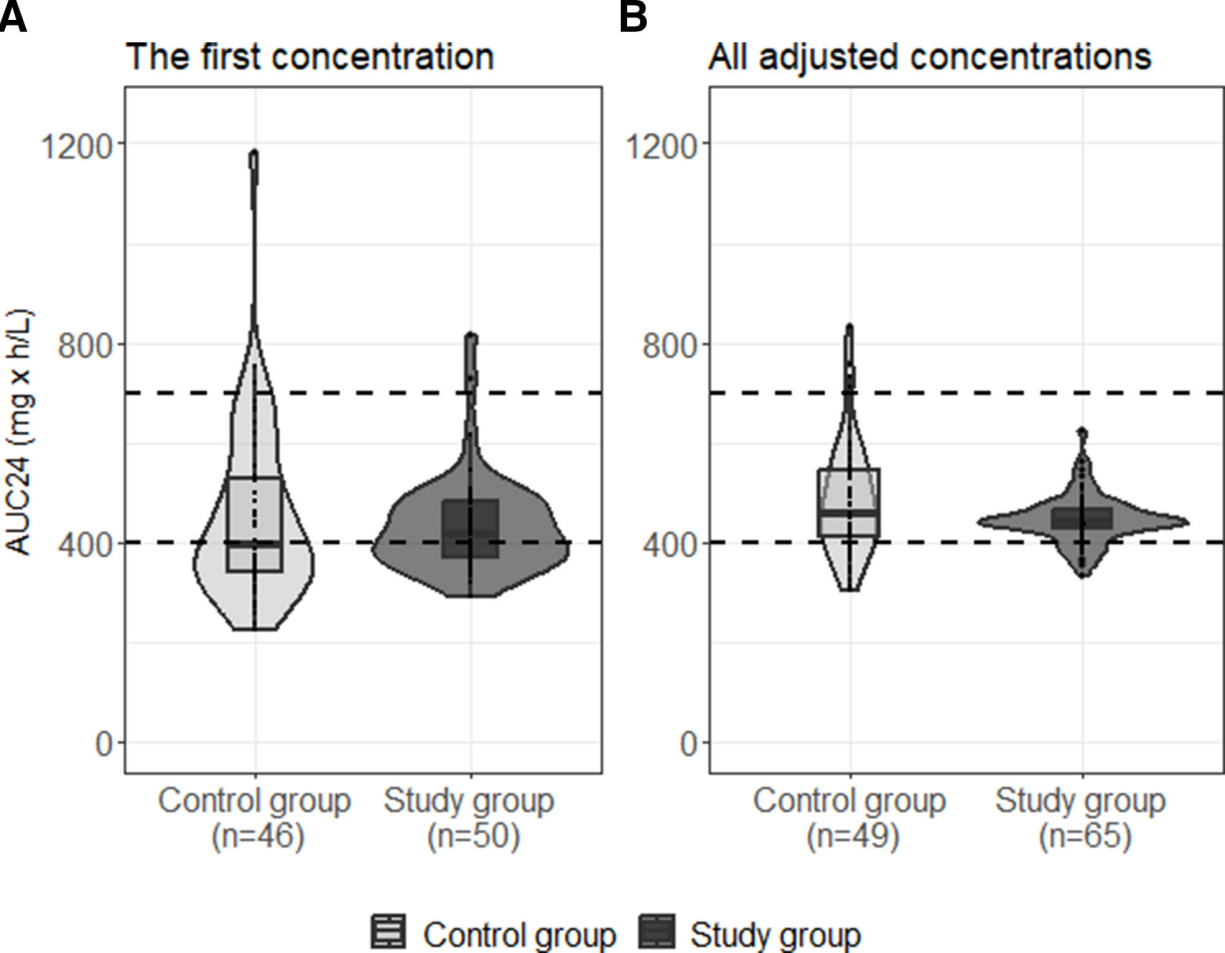
The violin plots and boxplots of the 24-h area under the time-concentration curve of vancomycin calculated based on (**A**) the first measured steady-state *C*_trough_ (SS-C_trough_) and (**B**) all SS-C_trough_ after adjusted doses in control (light gray) and study group (dark gray). The dashed lines show the target range of AUC/MIC 400–700 mg∙h/L.

After the first and all other optimized doses, the TA% *C*_trough 10-15 mg/L_ and TA% *C*_trough 8-17 mg/L_ were significantly greater in the study compared with the control group (Table 2). Sensitivity analyses were in line with the main analyses (Table 2). Of the AUC/MIC calculated based on the first SS-C_trough_, 53.1% (26/49) in the study compared with 43.5% (20/46) in the control group, but 84.4% (54/64) and 69.4% (34/49), respectively, when including all measured SS-C_trough_, were within the target range of 400–700 mg∙h/L (Fig. 2).

**TABLE 2:** Target attainment of measured steady-state trough concentrations in the range of 10–15, 8–17, and 5–20 mg/L and of sensitivity analysis, including in addition predicted steady-state trough concentrations for dosing intervals, where non-trough concentrations were available, after the first optimized and any adjusted dose^*a*^

	Control group	Study group	*P*-value
Final analysis including only measured *C*_trough_ values			
After the first optimized dose			
*C*_trough_ 10–15 mg/L	9/46 (19.6%)	25/50 (50.0%)	0.002
*C*_trough_ 8–17 mg/L	21/46 (45.7%)	38/50 (76.0%)	0.004
*C*_trough_ 5–20 mg/L	35/46 (76.1%)	45/50 (90.0%)	NS
After any adjusted dose			
*C*_trough_ 10–15 mg/L	18/49 (36.7%)	40/65 (61.5%)	0.01
*C*_trough_ 8–17 mg/L	31/49 (63.3%)	54/65 (83.1%)	0.01
*C*_trough_ 5–20 mg/L	45/49 (91.8%)	62/65 (95.4%)	NS
Sensitivity analysis including measured and predicted *C*_trough_ values			
After the first optimized dose			
*C*_trough_ 10–15 mg/L	18/69 (26.1%)	25/50 (50.0%)	0.007
*C*_trough_ 8–17 mg/L	33/69 (47.8%)	38/50 (76.0%)	0.002
*C*_trough_ 5–20 mg/L	53/69 (76.8%)	45/50 (90.0%)	NS
After any adjusted dose			
*C*_trough_ 10–15 mg/L	33/82 (40.2%)	41/73 (56.2%)	0.04
*C*_trough_ 8–17 mg/L	56/82 (68.3%)	62/73 (84.9%)	0.01
*C*_trough_ 5–20 mg/L	72/82 (87.8%)	70/73 (95.9%)	NS

^*a*^ Data are presented as *N* in target/*N* available (percent in target). NS, not significant (*P*-value > 0.05); *C*_trough_, trough concentration.

All patients survived. No one had acute kidney injury (12). Otoacoustic emission test was not passed in ≥1 ear in 9/45 (20%) and 5/60 (8.3%) patients in the study and control groups, respectively (*P* = 0.06). All adverse events in the study group are listed in Table S1.

Using MIPD software, we showed that MIPD significantly improved the attainment of *C*_trough_ (by 59% of the narrow target 10–15 mg/L and 38% clinically acceptable range 8–17 mg/L), outperforming that found in other neonatal studies, with similar side effect profile (4, 6, 7).

We considered both *C*_trough_ and AUC/MIC for the optimization target. Even if the correlation between *C*_trough_ and AUC/MIC is only moderate or poor, the surrogate marker, *C*_trough_ 10–15 mg/L should mostly ensure adequate exposure (AUC/MIC 400–700 mg∙h/L) of the drug (13, 14).

Although the predictive performance of the numerous published neonatal vancomycin popPK models varies widely, bias can be significantly reduced with the incorporation of a single TDM result already (9, 15). We included all available TDM data in each subsequent dose optimization, although studies have not found additional value in including more than two prior TDM points in MIPD dose optimization (9, 16).

Our study has some limitations. First, we used a historical control group that may bias the results due to differences in study populations (e.g., lower gestational age associated with a higher variability of PK and carrying higher risk of hearing disturbance). However, simulation and prospective intervention studies have shown improved TA% with the MIPD software (4, 6). Thus, conducting a randomized controlled trial of MIPD may raise ethical issues. Second, a large part of the study population and the population for the selection of the best-performing model in our MIPD tool development were recruited from one center, possibly affecting the generalizability of our results to other institutions. Third, dose optimization was performed by one researcher. Therefore, we may have missed some user-related real-life issues.

In conclusion, MIPD software appears to be a promising tool to improving PKPD target attainment of vancomycin therapy in neonates without undermining safety, especially when a narrow therapeutic window is targeted.

## SUPPLEMENTAL MATERIAL

Table S1 aac.01591-23-s0001.docx Adverse events in study group during the treatment and up to 7 days thereafter.

ASM does not own the copyrights to Supplemental Material that may be linked to, or accessed through, an article. The authors have granted ASM a non-exclusive, world-wide license to publish the Supplemental Material files. Please contact the corresponding author directly for reuse.
