# Supplementary material for: Prospective validation of a model-informed precision dosing tool for vancomycin treatment in neonates
Source: Antimicrob Agents Chemother. 2024 Apr 5;68(5):e01591-23. doi: 10.1128/aac.01591-23 (PMC11064528; doi:10.1128/aac.01591-23)
Supplement: Table S1 — Adverse events in study group during the treatment and up to 7 days thereafter. [file aac.01591-23-s0001.docx]

Supplementary material S1

Sample size was calculated based on the ratio of the control to the study group size 2:1, significance level α = 0.025, type II error rate β = 0.2 and the improvement of TA% C_trough 10-15_ from 25.3% (retrospective data from TUH between Oct 2015- Oct 2018) to 60%. According to the Fleiss formula, the required sample size was 100 participants in the control and 50 in the study group.

AUC/MIC (mg∙h/L) was calculated in NONMEM version 7.5 (ICON Development Solutions, MD, USA). The statistical analysis was conducted in R (version 4.1.0).

| **SAE- serious adverse events:**   - Septic shock: 1 patient (2%) - Cardiac post ligation syndromes: 1 patient (2%) - Severe hypocalcaemia: 1 patient (2%)   **pSAE-predictable serious adverse events**   - Persistent ductus arteriosus (PDA) medical or surgical closure: 6 patients (12%) with 7 episodes - New necrotizing enterocolitis (NEC II): 1 patient (2%) - New intraventricular haemorrhage (IVH): 1 patient (2%) - New infection   - Confirmed new systemic infection: 5 patients (10%)   - Suspected new systemic infection: 4 patients (8%)   - Pneumonia: 4 patients (8%)   - Conjunctivitis: 2 patients (4%)   - Omphalitis: 1 patient (2%)   **AE- adverse events**   - Anaemia of prematurity: 25 patients (50%) with 34 episodes (17 with 1 episode, 7 with 2 episodes, 1 with 3 episodes) - Electrolyte or glycose disorders: 10 patients (20%) - Skin problems at the site of vein cannula: 8 patients (18%) with 9 episodes - Worsening of breathing problems: 5 patients (10%) - Hemodynamic or volume disturbance (need for thrombocytes, albumin, plasma transfusion or inotropes): 4 patients (8 %) - Wrong dosing of vancomycin: 3 patients (6%) - Pastosity, need for a diuretic: 2 patients (4%) - Protein overload: 1 patient (2%) - Worsening of renal function (sCr increase): 1 patient (2%) - Nephrocalcinosis: 1 patient (2%) - Pneumothorax: 1 patient (2%) - Worsening of feeding problems: 1 patient (2%) - Osteopenia: 1 patient (2%) - Inguinal hernia: 1 patient (2%) - Dynamic ileus: 1 patient (2%) - Chemical ventriculitis (associated with IVH I): 1 patient (2%) |
| --- |

Table S1. Adverse events in study group during the treatment and up to 7 days thereafter
